# The roles of GTPase-activating proteins in regulated cell death and tumor immunity

**DOI:** 10.1186/s13045-021-01184-1

**Published:** 2021-10-18

**Authors:** Hua He, Jingjing Huang, Sufang Wu, Shiyao Jiang, Lu Liang, Yueying Liu, Wenbing Liu, Li Xie, Yongguang Tao, Yiqun Jiang, Li Cong

**Affiliations:** 1 The Key Laboratory of Model Animal and Stem Cell Biology in Hunan Province, Hunan Normal University, Changsha, 410013 Hunan People’s Republic of China; 2 School of Medicine, Hunan Normal University, Changsha, 410013 Hunan People’s Republic of China; 3 Department of Head and Neck Surgery, The Affiliated Cancer Hospital of Xiangya School of Medicine, Central South University, Changsha, 410013 Hunan People’s Republic of China; 4 Key Laboratory of Carcinogenesis and Cancer Invasion, Ministry of Education, Department of Pathology, Xiangya Hospital, School of Basic Medicine, Central South University, Changsha, 410078 Hunan People’s Republic of China

**Keywords:** GTPase-activating proteins, Regulated cell death, Tumor immunity

## Abstract

GTPase-activating protein (GAP) is a negative regulator of GTPase protein that is thought to promote the conversion of the active GTPase-GTP form to the GTPase-GDP form. Based on its ability to regulate GTPase proteins and other domains, GAPs are directly or indirectly involved in various cell requirement processes. We reviewed the existing evidence of GAPs regulating regulated cell death (RCD), mainly apoptosis and autophagy, as well as some novel RCDs, with particular attention to their association in diseases, especially cancer. We also considered that GAPs could affect tumor immunity and attempted to link GAPs, RCD and tumor immunity. A deeper understanding of the GAPs for regulating these processes could lead to the discovery of new therapeutic targets to avoid pathologic cell loss or to mediate cancer cell death.

## Introduction

The human Ras superfamily (monomeric GTPases) of small guanosine triphosphatases (small GTPases) comprises more than 150 members [1] and is usually divided into five main families: the Ras, Rho, Rab, Arf and Ran families [2]. They are associated with diverse cellular processes, including signal transmission, material transport and construction of the cytoplasmic skeleton [3]. Small GTPases have two different conformational states and cycle back and forth between them. In the activated state, they are bound to GTP, and the opposite is true for GDP. This state transition is managed by three regulators: guanine nucleotide exchange factors (GEFs), guanine nucleotide dissociation inhibitors (GDIs) and GTPase activating proteins (GAPs) [4]. Among these, GEFs are positive factors that activate GTPase by promoting binding to GTP, while GDIs and GAPs are both negative factors that inactivate GTPase by sequestering and hydrolyzing GTP, respectively [3, 4].

**Fig. 1:**
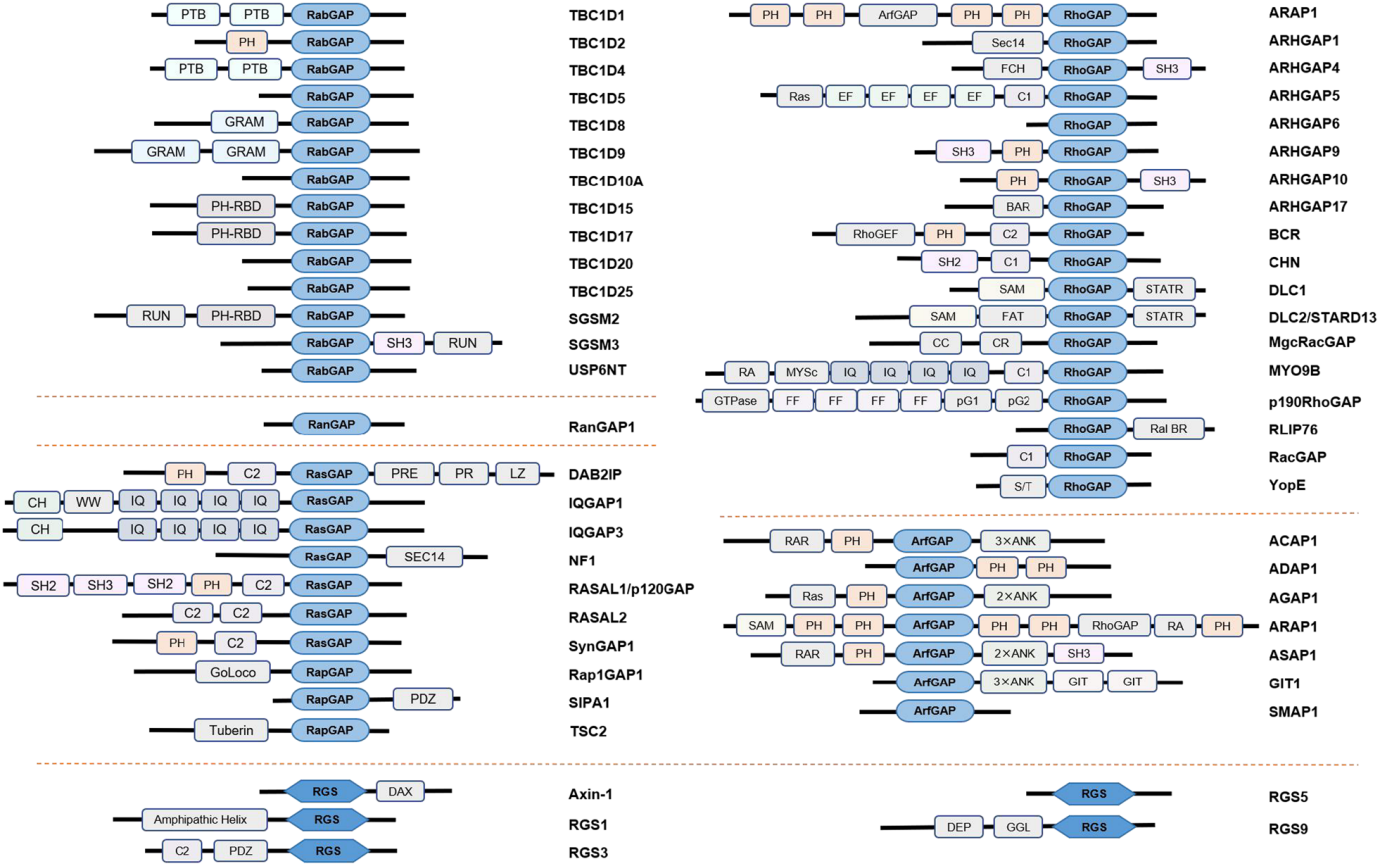
GAPs are multidomain proteins. GAPs have typical GAP active structural domains that interact with GTPase proteins, and other protein structural domains might also be present to play regulatory functions

GAPs are multiple structural domain proteins (Fig. 1) that range from 50 to 250 kDa in size [5]. Corresponding with the Ras superfamily of GTPases, GAPs can also be divided into five principal families: the Ras-GAPs, Rho-GAPs, Rab-GAPs, Arf-GAPs and Ran-GAPs families. In contrast to GAPs for the Ras superfamily, a class of GAPs, called regulators of G protein signaling (RGSs), acts on heterotrimeric G proteins [5, 6]. Once in the GDP-bound conformation, GAPs can generally terminate the corresponding downstream signaling cascades by hydrolyzing GTP. The GTP hydrolysis reaction is extremely slow, but GAPs can expedite the cleavage step by several orders of magnitude to increase the hydrolysis rate. During Ras-GTP hydrolysis, traditional GAPs insert the arginine finger or the asparagine thumb into the nucleotide-binding groove of the targeted GTPase to stimulate hydrolysis [7, 8], while RGS proteins directly bind to the active Gα subunits of the G-protein coupled receptor (GPCR) to induce hydrolysis [6].

Physiologically, cell death is a homeostatic mechanism that regulates and maintains the function and size of tissues and organs. Significantly different from accidental cell death (ACD), the consequences of external environmental disturbance, regulated cell death (RCD), is required for physiological or pathological nuisances that activate endogenous genetically encoded molecular structured signaling cascades and mechanisms, which can be interfered with by genetic or pharmacologic medicine. RCD can be classified into two master types: apoptotic and nonapoptotic. Apoptosis is the most common form of programmed cell death (PCD), while another major category of nonapoptotic RCD, which includes necroptosis, autophagy, mitotic catastrophe, pyroptosis, ferroptosis, methuosis, paraptosis, parthanatos, lysosome-dependent cell death, entosis and oncosis, is also gaining attention [9, 10]. These different types of cell death are distinct by the morphological changes and biochemical features caused by their death stimuli, but some forms of cell death are not completely independent of others, with certain intersections of molecular characteristics, such as apoptosis and autophagic cell death [11]. Irrespective of the rules of normal cell death, cancer cells survive when not supposed to, in large part, by producing relevant genetic mutations or epigenetic modifications that affect the transmission of cell death signals to circumvent RCD.

The regulation of GTPase activity by GAPs triggers a series of signaling changes, specifically in cell growth, proliferation, and death, and we framed what we can observe in terms of RCD and tumor immunity to explore the links among these three. In this review, we first will discuss the understanding of the molecular mechanisms of GAPs for different RCDs, illustrating with a large number of individual examples (Table 1), and finally, a major focus will be placed on the regulation of tumor immunity by GAPs. By summarizing this knowledge, we will further elaborate on the pathophysiological implications of GAP regulation of these processes and highlight promising cancer therapeutic approaches in light of these new findings.

## Apoptosis

Apoptosis is a form of PCD and is also termed ‘shrinkage necrosis’ [12] because of the morphological characteristics of chromatin condensation and cell shrinkage (pyknosis). In addition, its features include DNA fragmentation (karyorrhexis), apoptosome formation and membrane blebbing [9]. Two common signaling pathways induce apoptosis: one pathway is the intrinsic pathway, which is due to changes in mitochondrial membrane potential and outer membrane permeability and then promotion of the release of mitochondrial proteins such as cytochrome c, thereby activating caspase 3 and forming apoptosomes [13]. This process is regulated by the BCL-2 family of proteins, mainly proapoptotic proteins (BAX, BAK, BIM, PUMA and BID) and antiapoptotic proteins (BCL-2, BCL-XL and MCL1) [14]. The other pathway is the extrinsic apoptosis pathway that is initiated by the death-inducing signaling complex (DISC) and death receptors (cell membrane protein), such as Fas, tumor necrosis factor (TNF) receptors, and TNF-related apoptosis-inducing ligand (TRAIL) receptors, which ultimately activate the caspase protease family, the executors of cell apoptosis, and induce cell apoptosis [13, 15]. Apoptosis disorders are closely related to the occurrence and development of autoimmune diseases, neurodegenerative diseases and tumors. For example, cancer cells often have the characteristics of inhibiting apoptosis to ensure unlimited proliferation.

**Fig. 2:**
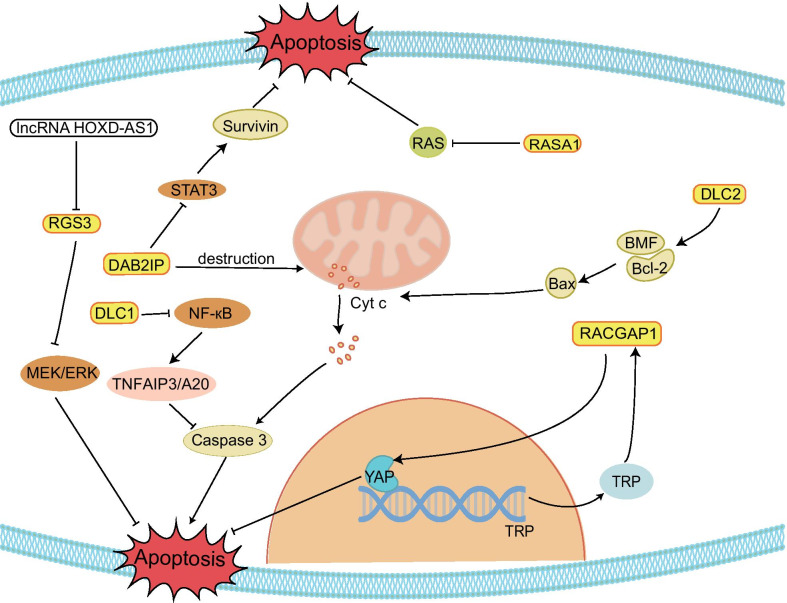
Examples of the involvement of GAPs in tumor cell apoptosis. Some GAPs can promote apoptosis in tumor cells by regulating apoptosis-related proteins and pathways and thus become collaborators of antitumor drugs. Some GAPs also exert apoptosis-inhibiting effects and thus promote tumor progression

Emerging studies have indicated that GAPs are closely associated with apoptotic progression (Fig. 2). Some GAPs can promote apoptosis of tumor cells to protect the organism. p120RasGAP, regulator of G-protein signaling 3 (RGS3), deleted in liver cancer 1 (DLC1), DOC-2/DAB2 interacting protein (DAB2IP), and STARD13 are typical examples because the five GAPs can influence the balance of antiapoptotic proteins and proapoptotic proteins and/or the corresponding signaling pathway to induce apoptosis. p120RasGAP (also known as RASA1), the classical GAP of the RAS, induces Ras-dependent tumorigenicity when its transcriptional regulation is repressed. Sorafenib, as a targeted agent in hepatocellular carcinoma (HCC), can induce apoptosis of tumor cells. Studies have shown that its important pathway upregulates the level of p120RasGAP for its therapeutic effect by promoting the phosphorylation of pituitary homeobox 1 (PITX1) to increase its expression and stability [16]. However, whether apoptosis can be successfully induced depends on the degree of signaling pathway activity. Caspase-3 is more mildly activated, which counteracts apoptosis and promotes cell survival by cleaving p120RasGAP into two fragments; its N-terminal fragment activates the PI3k/Akt pathway, and only the hyperactivation of caspase will promote apoptotic cell death [17, 18]. In HCC, the expression of RGS3 is influenced by the oncogenic lncRNA HOXD-AS1, which decreases the mRNA levels of RGS3 and activates the MEK/ERK signaling pathway to prevent apoptosis [19]. HOXD-AS1 also upregulates the expression of ARHGAP11A (a RhoGAP) and leads to the induction of metastasis by serving as a competing endogenous RNA (ceRNA) and repressing miR19 [19]. Similar to HOXD-AS1, the STARD13 (DLC2, a RhoGAP) 3’UTR acts as a ceRNA and increases Bcl-2 modifying factor (BMF) expression by competitively binding with miR-125b in breast cancer. Meanwhile, the STARD13 3’UTR could promote the interaction of BMF/Bcl-2 to release Bax and cytochrome c to activate the intrinsic pathway of apoptosis [20]. DLC1 and DAB2IP directly affect the corresponding pathway and target protein to induce apoptosis. For example, DLC1 (a RhoGAP) deregulates the expression of TNFAIP3/A20 and upregulates the expression of BCL211/BIM and caspase-3 to induce cell death by inactivating NF-кB signaling in angiosarcoma [21]. The DAB2IP effect on promoting apoptosis involves multiple signaling pathways in cancer [22]. In prostate cancer (PCa), DAB2IP has a dual role in influencing apoptosis. First, DAB2IP directly interacts with STAT3 and inhibits its phosphorylation (tyrosine 705 and serine 727) and transactivation, thereby disturbing the balanced expression of pro-apoptotic genes (Bax) and anti-apoptotic genes (surviving, Bcl-2 and Bcl-xL) and promoting apoptosis. Second, DAB2IP activates intrinsic pathways, including disruption of the mitochondrial membrane potential and release of cytochrome c, Omi/HtrA2 and Smac, ultimately activating the caspase cascade [23].

RACGAP1 can promote the metastasis and development of cancer by inhibiting apoptosis. RACGAP1 acts on small G proteins of the Rho family, stimulating GTP hydrolysis and regulating CDC42 and RAC1. The expression and stability of RACGAP1 are influenced by STAT3 and epithelial cell transforming sequence 2 (ECT2). In HCC, STAT3, a transcription factor of RACGAP1, can upregulate the expression of RACGAP1, and then, RACGAP1 reduces the Hippo signaling pathway through the accumulation of F-actin to activate the transcription coactivator yes-associated protein (YAP). With YAP, the transcription of the nucleoporin translocated promoter region (TPR) is upregulated. TPR in turn can regulate the phosphorylation and localization of RACGAP1 in the central spindle. As a result, apoptosis is inhibited while the growth of tumor cells is promoted [24]. ECT2, the catalytic agent of guanine nucleotide exchange on small GTPases [25], interacts with RacGAP1. In HCC, on the one hand, ECT2 promotes RacGAP1 protein stability, and on the other hand, RacGAP1 promotes ECT2-mediated RhoA activation and HCC cell metastasis [26]. In basal-like breast cancer (BLBC), knockdown *RACGAP1* cells have also been shown to fail in cytokinesis and cause the initiation of apoptosis [27].

Certainly, GAPs also play a significant role in other apoptosis-related diseases except for cancer. Untimely and inappropriate apoptosis will elevate the occurrence rate of cardiovascular disease. Seventy percent of capillary malformation-arteriovenous malformation patients present with inactivated mutations in the *RASA1* gene. Most likely, based on the function of the cleaved N-terminal fragment of RASA1 in mediating anti-apoptosis, RASA1 deficiency leads to apoptosis of lymphatic vessel (LV) endothelial cells, triggering the impaired formation of LV valves [28]. In addition, RGS5 not only coordinates the activity of proapoptotic proteins, antiapoptotic proteins and caspase-3 but also inhibits the JNK1/2 and p38 signaling pathways to inhibit the apoptosis of cardiomyocytes, which exists in myocardial ischemia reperfusion [29]. Unnecessary apoptosis is also related to neurological diseases and optic neuropathy. Researchers suggest that the overexpression of DAB2IP, which has the new name apoptosis signal-regulating kinase 1-interacting protein-1, can promote the development of Alzheimer's disease by mediating β-amyloid-induced apoptosis of cerebral endothelial cells, while the overexpression of TBC1D17 will promote retinal cell death to achieve optic neuropathy [30, 31].

In summary, the aforementioned GAPs interact with their target protein or signaling pathways to activate or inhibit apoptotic signaling pathways and influence apoptosis, thereby affecting the development of the disease. Continuously, scholars have paid strong attention to the mechanisms and therapeutic strategies of tumors. Here, we introduce some GPAs that affect apoptosis to reveal the pathological process and improve the therapeutic effect of tumors. Of course, GAP research also reveals the special mechanisms of other pathological processes to give us a better understanding of the design and help us to develop an effective treatment for special targets.

## Non-apoptotic RCD

**Fig. 3:**
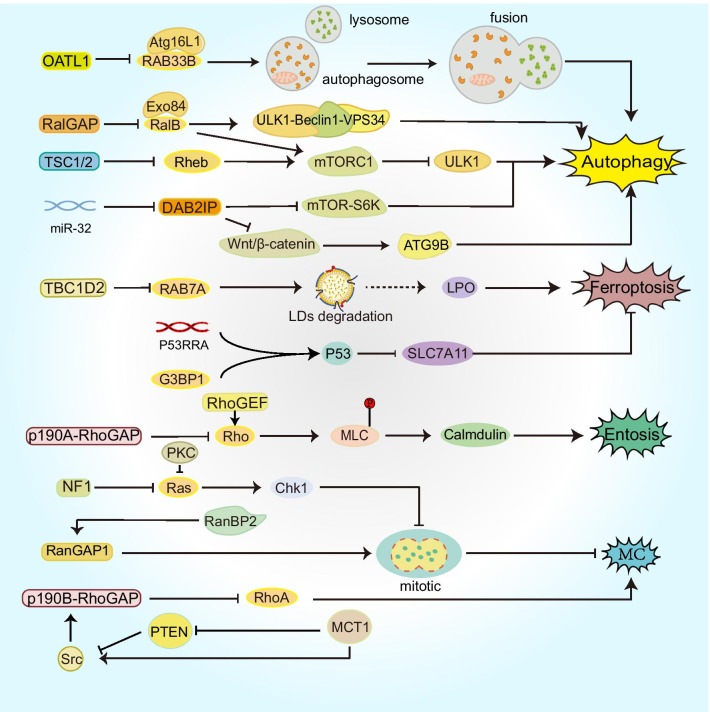
Signaling pathways of GAPs that affect nonapoptotic RCD. GAPs regulate vesicle transport and autophagosome maturation during autophagy and are involved in mTOR-related pathways. In addition, GAPs regulate ferroptosis, entosis, MC and other RCDs

This section covers not only autophagy-dependent cell death to explain the association with GAPs but also novel forms of cell death, such as ferroptosis, pyroptosis and other informal types (Fig. 3).

### Autophagy-dependent cell death

To maintain our physical homeostasis and health, it is necessary to activate autophagy to eliminate the redundant and harmful components of the cell. Autophagy is an important, conserved and normal cellular process that is always divided into several steps: induction of phagophores, formation of autophagosomes and autolysosomes, and degradation and recirculation of luminal contents. The main characteristics are special membrane structures, including phagophores, autophagosomes and autolysosomes. Autophagy is considered to be a cell survival mechanism, but when autophagy is overactivated beyond the cell's capacity, it leads to cell death, called autophagy-dependent cell death (ADCD). The identification of ADCD requires features of an increased rate of autophagic activity and exclusion of cell death due to other forms, and it can be modified by genetic and/or pharmacological inhibition of autophagy factors [32]. However, the concept of ADCD is still highly controversial. On the one hand, the existence of crosstalk between autophagy and other RCDs, such as apoptosis, makes it difficult to define ADCD as an independent cell death process only by relevant molecular and morphological markers, and on the other hand, a threshold to classify lethal and nonlethal autophagy is difficult to determine [33, 34]. The role of autophagy in tumors could be two-sided. Although the loss of autophagy promotes tumor progression in mouse models, more evidence demonstrates that autophagy can suppress tumor-specific inflammatory responses and assist tumor cell metabolic activities in a nutrient-limited microenvironment, promoting tumor growth [35, 36]. It has been shown that some anticancer drugs, such as resveratrol and arsenic trioxide, can induce ADCD [37–39], and in addition, ADCD occurs in oncogenic Ras-expressing cells in the absence of other cotransformed genes [40], but its role in various tumors remains to be explored. Therefore, we provide only a limited introduction to the role of GAPs in autophagy here.

Autophagosomes are morphological markers of autophagy, while autophagy (ATG)-related proteins are key to autophagosome formation and are molecular markers of autophagy. RAB GTPases can control the transport of intracellular vesicles [41] and designate autophagosome maturation [42]. Approximately 10 RAB proteins have definite functions in autophagy [43]. Therefore, RABGAPs, including TBC (TRE2-BUB2-CDC16) domain-containing RABGAPs, are also involved in autophagy. RAB33B affects the formation of autophagosomes by recruiting the Atg12-Atg5-Atg16L1 complex into phagocytes, and Atg16L1 is the binding protein of RAB33B [44]. One study showed that OATL1 is a GAP acting on RAB33B, and its overexpression can delay autophagosome maturation by regulating the fusion between autophagosomes and lysosomes [45]. RalA/B (RAS like A/B), a member of the Ras GTPase family, is also a key regulator of vesicle transport [46]. In the mammalian cell model, RalB and its effector protein Exo84 together induce ULK1-Beclin1-VPS34 assembly, which is required for autophagosome formation. Under experimental conditions lacking nutrient limitation, a decrease in RalGAP can activate RalB and induce an increase in autophagy [47]. In another experiment using Drosophila as a model, researchers found that Ral GTPase regulates autophagy in the context of PCD [48], which could be considered to be ADCD.

The mechanistic target of rapamycin (mTOR) integrates growth factors and nutrient signals to inhibit autophagy. mTORC1, which is a signaling complex with mTOR as the major component, promotes phosphorylation of ULK1 (unc-51-like kinase 1) in the presence of sufficient nutrients [49]. Under the regulation of AKT and AMPK signaling kinases, tuberous sclerosis complex 1/2 (TSC1/2) acts as the GAP of Rheb (the Ras homolog enriched in the brain) to inhibit the formation of GTP-bound Rheb and participates in the regulation of the Rheb-mTORC1-ULK1 signaling pathway to promote autophagy [49–51]. Tsc1/2 deficiency is responsible for the development of tuberous sclerosis complex (TSC), an autosomal dominant disorder that predisposes patients to the development of tumors of multiple organ systems [52]. Therefore, defective autophagy in TSC could lead to the accumulation of autophagic substrates, including abnormal proteins and organelles, within the cell, promoting tumorigenesis. Studies have also shown that the deletion of RalGAP induces an increase in mTORC1 activity, leading to a decrease in autophagy. Meanwhile, in pancreatic cancer, RalGAP suppresses tumor cell invasion through mTORC1 signaling [53]. Autophagy increases the resistance of tumor cells to chemotherapy and radiotherapy. Temozolomide (TMZ) for the treatment of glioblastoma (GBM) is prone to induce autophagy and could make tumor cells resistant to the drug. DAB2IP was found to negatively regulate ATG9B expression through the Wnt/β-catenin signaling pathway, thereby inhibiting TMZ-induced autophagy and increasing drug sensitivity in GBM cells [54]. In addition, DAB2IP has also been shown to be a negative regulator of autophagy-related radiation resistance in PCa. As an upstream regulator of DAB2IP, miR-32 downregulates the protein level of DAB2IP by targeting its 3'-UTR and inhibiting its translation [55]. Subsequently, the downstream mTOR-S6K pathway is activated, but autophagy activity is enhanced, which could be the result of negative feedback inhibition of Akt [56], ultimately enhancing the radiation resistance of PCa cells [55, 57].

Some GAPs influence the nervous system by regulating autophagy. SIPA1L2, a Rap GTPase-activating protein, regulates the process of neurotransmitter liberation, which is linked to TrkB/Rap1 signaling and amphisomes that are the fusion organelles of TrkB-late endosomes with autophagosomes [58], while others, including TBC1D5 and TBC1D15, are associated with motor neuron disease, and these GAPs cause the disorder-degradable process of autophagy and the aggregation of toxic proteins [59–63]. SGSM3/RABGAP5 and TBC1D10A both inactivate the corresponding GTPases to terminate autophagy and have effects on the immune system when autophagy eliminates pathogens and damages the organelles of cells [64, 65]. The absence of GAPs could lead to genetically heterogeneous autosomal diseases. For example, deletion of the TBC1D20 protein can increase the accident rate of Warburg Microsyndrome 4, which is an autosomal disorder and possesses abnormal eye, brain and genital functions [66]. Autophagy is also an intrinsic mechanism to maintain metabolism and recycle nutrients during starvation or stress. TBC1D5 binds and sequesters LC3^+^ autophagic compartments and increases glucose transporter GLUT1/Slc2a1 expression on the plasma membrane, facilitating glucose uptake and glycolytic flux [67].

In conclusion, most GAPs downregulate the corresponding GTPase activity to directly regulate autophagy to influence our physical functions, but few serve as effectors to indirectly regulate autophagy to achieve that goal. Autophagy is closely related to physical homeostasis and health. Significantly, GAPs influence the process of autophagy. Unfortunately, ADCD itself has many unresearched areas, and as a result, there are few studies on ADCD and GAPs. We can only infer the possible role of GAPs in ADCD from the connection between autophagy and GAPs. Therefore, further studies are necessary to give us a better understanding of how GAPs regulate ADCD in physiological and pathological situations, correctly understand pathological development and find therapeutic targets.

### Ferroptosis

Ferroptosis is a novel oxidative RCD in which the consequences are accumulated by lethal iron dependence of lipid hydroperoxides [68]. Its scientific observation initiates the experiment of erastin-induced selective cell death in 2003, and the term “ferroptosis” was coined in 2012 [69]. Afterward, scholars generated a surge in ferroptosis research. The unique feature of its morphology is mitochondrial changes that include small size, alteration of membrane densities, reduction or vanishing of mitochondrial crista, and rupture of outer membranes [70]. Ferroptosis is associated with a variety of diseases, including acute kidney injury, cancer, and cardiovascular disease. Part of the induction of ferroptosis is RAS-dependent [71]. In Ras mutant cancer cells, blocking the RAS-RAF-MEK pathway inhibits ferroptosis induced by erastin, which is an antitumor drug that promotes cell death [72]. However, relatively little is known about the connection between GAPs and ferroptosis.

A large number of molecular markers and pathways have been described for autophagy as possible processes of ferroptosis [9, 73]. GTPases and GAPs that have a role in autophagy could also be regulators of ferroptosis. RAB7A is involved in autophagy-induced degradation of lipid droplets (LDs), and the accompanying lipid peroxidation exacerbates ferroptosis [74]. Accordingly, TBC1D2, as a negative regulator of RAB7A, could regulate ferroptosis in a RAB7A-dependent manner [75]. G3BP1 (Ras-GTPase-activating protein-binding protein 1) is involved in the adjustment of the Ras signaling pathway. The process of it-induced cell death is linked to the long noncoding RNA P53RRA, which is regulated by LSH and p53. During that process, nucleotides 1 and 871 of P53RRA directly interact with the RNA recognition motif interaction domain of G3BP1 (aa 177–466), forming the P53RRA-G3BP1 complex. In the cytoplasm, the P53RRA-G3BP1 interaction displaces p53 from a G3BP1 complex, leading to the redistribution of p53 through p53 transfer from the cytoplasm to the nucleus, which activates the p53 signaling pathway and influences the expression of several metabolic genes, such as TIGAR and SLC7A11, eventually causing cell cycle arrest, which leads to apoptosis and ferroptosis [76].

### Pyroptosis

Pyroptosis is a type of inflammatory RCD that is an innate immune mechanism to resist pathogen invasion and maintain physical homeostasis [77]. Caspase-1/4/5/11 activation is induced by some inflammasomes, which increase the cleavage rate of gasdermin D and secrete mature inflammatory cytokines, such as interleukin-18 and interleukin-1β [78]. Its features are DNA fragmentation, cell swelling, and bubbles that ultimately rupture the plasma membrane.

The connection between pyroptosis and GAPs is reflected in the cell death induced by certain microorganisms. YopE is a type of *Yersinia* outer protein (Yops) and can act as the host GAP of Rho GTPase by hydrolyzing GTP-bound Rho GTPase in a noncovalent manner in *Yersinia*. During *Yersinia* infection and cell death induction, YopE has another collaborator, YopT, a cysteine protease that covalently decomposes the C-terminus of Rho GTPase, therefore leading to Rho GTPase dissociation and inactivation. Although YopE and YopT are essentially different from Rho GTPase inactivation, both are Rho-modifying toxins that influence host cell physiology and evade immune responses. This process is directly induced in a manner that dephosphorylates the active Ser205 and Ser241 sites of pyrin and forms a pyrin inflammasome, ultimately leading to pyroptosis [79].

### Entosis cell death

In 2007, researchers described the nonapoptotic cell death process entosis to account for the cell-eating phenomenon observed between tumor cells [80, 81]. When living cells are consumed by the same or different types of cells, a “cell within a cell” structure occurs, resulting in the death of internalized cells (entotic cells). Dying entotic cells do not have the morphological and molecular characteristics of apoptosis but exhibit autophagy dependence, with lysosome and vacuolar membrane autophagy protein-dependent fusion inducing entosis [82, 83].

Cell adhesion and cytoskeletal rearrangement are key processes in entosis and cannot be deficient in epithelial cadherin and Rho-ROCK signaling [80]. The recruitment of p190A RhoGAP at cell–cell junctions inhibits the activity of the Rho pathway, leading to a decrease in myosin light chain phosphorylation, which reduces actomyosin contraction and suppresses calmodulin levels. Due to the polarized distribution of p190A RhoGAP, the contraction of actin at the distal end of cell adhesion is significantly higher than that at the cell adhesion site [84]. In addition, Rho is activated by RhoGEF at the distal end of cell adhesion [85]. Therefore, RhoGAP and RhoGEF act separately for Rho but synergistically for the induction of entosis.

### Mitotic catastrophe

Mitotic catastrophe (MC) is a type of abnormal mitotic cell death that is also an effective anticancer mechanism and therapy [86]. Its morphological characteristics are unique nuclear alterations that usually exhibit multinucleation and/or micronucleation [87]. To be precise, MC is not a type of RCD because MC, like autophagy, does not necessarily cause cell death, and thus, the Nomenclature Committee on Cell Death 2018 recommends using the term mitotic death as the name of this type of death [10]. Moreover, studies have shown that the ultimate fate of most MC cells is intrinsic apoptosis [10, 88], with differences and connections between the two.

Three types of GAPs are linked with aberrant mitosis: RasGAP NF1, p190RhoGAP and RanGAP. Mutations in *NF1* can activate RAS-related downstream signaling pathways. In this case, coordination of other signaling pathways, such as PKC-related pathways, is needed to regulate the cellular disturbance of RAS overactivation and needed to ensure cell survival. Under Nf1-deficient conditions, the suppression of endogenous protein kinase C (PKC) most likely cooperates with the Akt (one of the downstream effectors of aberrant Ras) pathway to activate Chk1, prolonging mitotic arrest and subsequently causing apoptosis via MC [89]. Overexpression of multiple copies in T-cell malignancy 1 (MCT-1) confronts the presentation of the PTEN gene and negatively influences the stability and functional activity of its proteins, activating phosphoinositide 3 kinase/AKT signaling. In addition, MCT-1 downregulated p190RhoGAP and upregulated the expression of p190B, which binds Src, interacts with MCT-1 and activates Src/p190B signaling. In the end, the increased presentation of MCT-1 and the inhibited PTEN synergistically augment the Src/p190B pathway, which causes the depression of RhoA activity and enhances the occurrence rate of MC [90]. In contrast to the description of the above GAPs, in addition to the kinetochore and spindle localization of RanGAP1 being influenced by importin β1, which is a regulator involved in the vector of the main interphase nuclei and mitotic progression, RanGAP1 sumoylation is also related to importin β1 and shows a positive correlation. The mechanism is most likely that RanBP2 directly interacts with the N-terminus of importin β1, sequesters endogenous RanBP2, decreases it and importin β1, and diffuses both, leading to abnormal spindle formation and impaired chromosome alignment, which ultimately causes cell death [91]. In summary, the abnormal regulation of the corresponding GTPase protein activity by GAPs could disrupt the normal signal cascade and finally increase the rate of MC accidents.

**Fig. 4:**
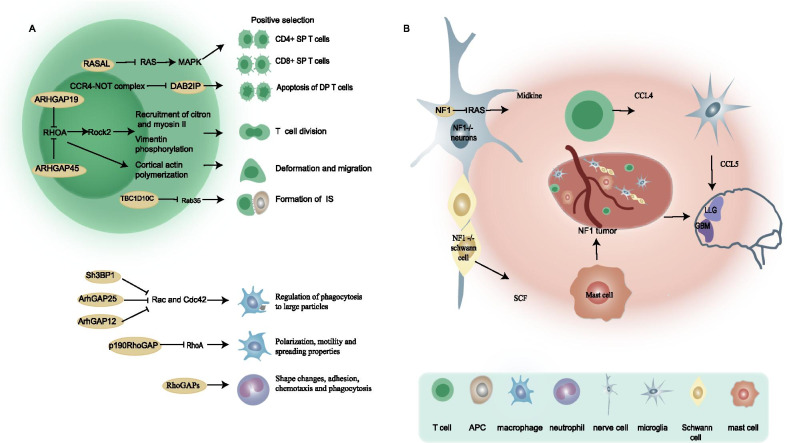
Role of GAPs in immune activity. **a** GAPs are essential for maintaining normal immune cell activity. **b** NF1 deficiency participates in the formation of the relevant tumor immune microenvironment

### Methuosis

Methuosis is a unique form of RCD, and its special character is vacuolization, the accumulation of vesicles (single membrane and from macropinosomes, distinguishing the structure of double membranes of autophagosomes) and eventually plasma membrane rupture [92]. Methuosis is closely associated with the Ras signaling pathway (continuous activation), which is clearly characterized in GBM and gastric carcinoma [93].

GIT1 serves as GAP to inactivate Arf6 by hydrolyzing GTP to influence methuosis. In that process, hyperactive H-Ras activates the Rac1 GEF, increasing the amount of Rac1-GTP. Micropinocytosis enhanced by the activation of Rac1 and clathrin-independent endocytosis (CIE) obtain some features of late endosomes (Rab7 and LAMP1). Meanwhile, there is a feedback mechanism in which hyperactive Rac1 enables Rac1-GTP to directly interact with GIT1, decreasing the activity of Arf6, impairing the recycling of CIEs and failing to fuse with lysosomes. Finally, these consequences lead to the accumulation of CIE, and late endosomal vesicles merge with one another, thus forming larger fluid-filled cytoplasmic vacuoles, which ultimately rupture the plasma membrane and cause cell death [94, 95]. However, the above result contradicts Shliom and colleagues’ finding that Arf6 (Q67 L) activity promotes vacuole formation in cells that express H-Ras (G12V) [96]. The most appropriate explanation for this phenomenon is that they influence the vacuoles that arise from different stages of the endocytosis pathway [94].

**Table 1:** The roles of GAPs in RCD

GAPs	RCD	Effect	Target	Associated physiology and pathology	References
p120RasGAP (RASA1)	Apoptosis	up/down	RAS, N-terminal fragment	Hepatocellular carcinoma, capillary malformation-arteriovenous malformation	[16, 28]
RGS3	Apoptosis	up	MEK/ERK	Hepatocellular carcinoma	[19]
STARD13	Apoptosis	up	Bax	Breast cancer	[20]
DLC1	Apoptosis	up	NF-кB	Angiosarcoma	[21]
DAB2IP	Apoptosis	up	STAT3, β-amyloid	Prostate cancer, Alzheimer's disease	[23, 30]
RACGAP1	Apoptosis	down	Hippo signaling pathway	Hepatocellular carcinoma	[24]
RGS5	Apoptosis	down	JNK1/2 and p38 signaling pathways	Cardiomyocytes	[29]
TBC1D17	Apoptosis	down	Rab12	Optic neuropathy	[31]
OATL1	Autophagy	down	RAB33B	Autophagosome maturation	[45]
TSC1/2	Autophagy	up	Rheb-mTORC1-ULK1 signaling pathway	Tuberous sclerosis complex	[51]
RalGAP	Autophagy	down/up	RalB, mTORC1	Autophagosome formation	[47, 53]
DAB2IP	Autophagy	down	Wnt/β-catenin signaling pathway, mTOR-S6K pathway	Glioblastoma, prostate cancer	[54, 55, 57]
SIPA1L2	Autophagy	down	TrkB/LC3b	Neurotransmitter liberation	[58]
TBC1D5	Autophagy	down	Rab7a	Neurodegenerative diseases, glucose uptake	[61, 67]
TBC1D15	Autophagy	down	RAB7	Neurodegenerative diseases	[62, 63]
SGSM3/RABGAP5	Autophagy	down	RAB5	Ehrlichia infection	[64]
TBC1D10A	Autophagy	down	Rab35-NDP52	Autophagosome formation	[65]
TBC1D20	Autophagy	down	RAB1B	Warburg Micro syndrome 4	[66]
TBC1D2	Ferroptosis	down	RAB7A	Lipid degradation	[75]
YopE	Pyroptosis	up	Rho GTPase	Yersinia infection	[79]
p190A RhoGAP	Entosis	up/down	Rho-ROCK signaling	Cell adhesion and cytoskeletal rearrangement	[84]
NF1	Mitotic catastrophe	up	RAS	Mitotic arrest	[89]
p190RhoGAP	Mitotic catastrophe	up	RhoA	Neoplastic multinucleation	[90]
RanGAP1	Mitotic catastrophe	up	Mitotic localization	Abnormal spindle formation	[91]
GIT1	Methuosis	up/down	Arf6	Vacuoles formation	[94–96]

## GAPs regulate tumor immunity

### RCD and immunity are closely related

RCD was initially thought to be an immune-tolerogenic event, especially apoptosis [97]. However, later evidence and the introduction of the concept of immunogenic cell death (ICD) have gradually established the role of immune activity in RCD. Actually, ICD is not an independent mode of death, and it refers to a type of RCD that has the presence of adaptive immunity driven by activation of cytotoxic T lymphocytes (CTLs) in response to stress-induced cell death [97, 98]. The development of ICD is a complex process in which the existence of antigens not covered by central tolerance in dead cells and the exposure and release of damage-associated molecular patterns (DAMPs) are key components, referred to as antigenicity and adjuvanticity, respectively [98]. DAMPs promote the recruitment and maturation of antigen-presenting cells (APCs), triggering a CTL-dependent immune response [99]. Some conventional chemotherapeutic agents, oncolytic viruses, targeted anticancer agents, specific radiotherapy modalities, and other factors could be inducers of ICD [100, 101]. Based on this finding, in 2013, researchers suggested that combinations of ICD inducers with other immunomodulators could lead to effective antitumor effects [99], and subsequent studies have confirmed that monoclonal antibodies targeting classically inhibited immune checkpoints, such as cytotoxic T-lymphocyte-associated antigen 4 (CTLA-4), programmed cell death-1 (PD-1) and its corresponding ligand PD-L1, are good collaborators for ICD [102–104]. Recently, cancer immunotherapy combined with nanotechnology to induce ICD has also shown new prospects [105, 106]. Of course, other RCDs are not the end of the cell but could be the beginning of an immune response or even ICD [107]. Moreover, these RCDs are also engaged in antitumor immunity [108, 109]. For example, T cells and ferroptosis mediate each other in tumors. Immunotherapy-activated CD8 + T cells enhanced lipid peroxidation in tumor cells, which in turn contributed to the antitumor efficacy of immunotherapy with increased ferroptosis [110]. This evidence is sufficient to demonstrate that RCD is inextricably linked to immune activity and immunotherapy.

### GAPs contribute to the immune microenvironment

The formation and basal function of multiple immune cells are influenced by GAPs (Fig. 4A). T cells are the mainstay of antitumor immunity. Immature double-positive (DP) thymocytes are partially differentiated into CD4 + or CD8 + single-positive (SP) T cells after positive selection, whereas the other DP T cells undergo apoptosis. The mechanism by which the Ras-MAPK pathway regulates this process has been well studied [111, 112]. In RASA1-deficient thymus, DP cells have increased susceptibility to apoptosis, but the increased CD4 SP to DP ratio suggests that RASA1 deletion promotes positive selection and could be associated with Ras-MAPK signaling pathway activation [113]. In addition, given the proapoptotic effect of DAB2IP, the CCR4-NOT complex downregulates DAB2IP to participate in the positive selection of thymocytes [114]. Interestingly, another study showed that NF1 promotes positive selection of thymocytes in female HY TCR Tg mice, but the mechanism is unclear [115]. Another example of T cell regulation is that ARHGAP19 coordinates the cytoskeletal remodeling required for T lymphocyte division and controls chromosome segregation by regulating RhoA [116]. ARHGAP45 could regulate RHO to orchestrate changes in the cytoskeleton of naive T cells, increase their deformation and migration to lymph nodes (LNs), and promote thymic seeding of T cell progenitors [117]. In addition, Rab35 and its GAP EPI64C (TBC1D10C) are required in the formation of immunological synapses (ISs), which are a part of the T cell-APC interaction [118]. Macrophages play a key role downstream of the immune response by engulfing dead cells. Previous studies have characterized the Rho GTPase members Rac1 and Cdc42 as molecular switches that control actin cytoskeleton tissue to regulate Fc receptor-mediated phagocytosis [119, 120]. Sh3BP1, ArhGAP12 and ArhGAP25 cooperatively inactivated Rac and Cdc42 in time and space, thus ending the phagocytosis of macrophages to large particles such as apoptotic cells [121]. Macrophage polarization, motility and cell spreading properties are associated with RASA1-mediated regulation of p190RhoGAP translocation [122]. Another RhoGAP myosin Myo9b deletion in macrophages was shown to result in altered cell morphology and impaired migratory capacity [123]. The role of the RhoGAP family in neutrophils is more extensive, mainly involving neutrophil shape changes, adhesion, chemotaxis and phagocytosis, as reviewed by Roland Csépányi-Kömi et al. [124].

With the engagement of the tumor microenvironment, tumor cells can escape the surveillance of the immune system and thus survive immune attacks during the development process. *NF1,* encoding neurofibromin, is a good example to illustrate the role of GAPs in the tumor immune microenvironment (Fig. 4B). Neurofibromin is a GTPase-activating protein that downregulates RAS activity, and thus, mutations in *NF1* can activate RAS-related downstream signaling pathways. Neurofibromatosis type 1 (NF1) is a genetic disorder of the nervous system caused by the loss of activity of the neurofibromin protein GAPs [125]. Immune cells such as infiltrating inflammatory mast cells are a component of NF1, and mutations of the *NF1* gene in immune cells are also essential for this disease [126]. Researchers designed NF1^flox/−^; Krox20-Cre mice with *NF1*^*−/−*^ Schwann cells and *NF1*^+/−^ mast cells and found that mice with Schwann cell proliferation as well as massive mast cell infiltration developed plexiform neurofibromas compared to control mice. This finding demonstrates the fact that the haploinsufficiency of *NF1* mast cells creates an *NF1*^+/−^ immune microenvironment that favors tumors [127]. In addition, NF1^−/−^ Schwann cells enhance Nf1 haploinsufficiency mast cell migration by stem cell factor (SCF) and degranulation through c-kit-mediated activation of the PIK-3 pathway [128–130]. Compared to normal people, NF1 patients are prone to developing tumors of the central nervous system. In low-grade glioma (LGG), researchers have discovered a key neuroimmune axis, which suggests that *NF1* mutant neurons produce midkine to induce T cells to activate microglia to produce CCL5, a factor that promotes the growth of LGG [131]. Similar results were found in GBM. A recent study showed that tumor models with codeletion of *Nf1* and *Pten* and overexpression of *EGFRVIII* had the ability to escape immune clearance and a high degree of immunosuppressive microenvironment, and *Nf1* loss was the key event [132]. Interestingly, although the incomplete mutation of *NF1* alleles is a driver of tumors, some researchers present a contradiction in that the absence of NF1 in T cells could increase T cell activity to enhance the physical immune monitoring mechanism of the tumor and inhibit malignant migration. Consistent with this finding, the clinical phenomenon of NF1 patients in which most tumors associated with NF1 are nonmalignant remodels our recognition of *NF1* gene mutations [133]. In addition, studies have shown that the Ras protein activator-like 1 protein (Rasal1) negatively regulates the P21Ras-ERK pathway in T cells, thereby inhibiting the activation of T cells to reduce the antitumor immunity of T cells, while RASAL1 knockdown was shown to enhance the antitumor activity of T cells in B16 melanoma and EL-4 lymphoma [134]. As the GAP of G proteins, the RGS family is involved in the regulation of immune activity in many ways and has the potential for targeted immunotherapy [135]. Recent studies have shown that RGS1 inhibits the transport of Th1 cells and CTLs to tumors, facilitating the formation of 'cold tumors' in breast cancer and impairing antitumor immunity [136]. Meanwhile, mouse experiments demonstrated that transfer of tumor-specific CTLs with RGS1 knockdown in combination with PD-L1 could be a promising immunotherapeutic strategy for breast cancer [136].

## Conclusions and perspectives

Research on GAPs in diseases, especially cancer, has increased in recent years. Some GAPs could be influential factors in cancer cell proliferation, migration, drug resistance, and malignant transformation and could even be new therapeutic targets and prognostic markers for cancer. The signaling pathways associated with RCD could be tuned by GAPs during this process. The most typical example is the inhibition of RAS-related pathways by RASGAPs to regulate the apoptotic process in cancer cells. In fact, studies on the regulation of tumor immunity by GAPs are limited. Here, by summarizing the broad role of GAPs in regulating RCD, we speculate that it could be possible that GAPs are present in RCD-related immune activity or, more precisely, in ICD-induced antitumor immune responses. In addition, GAPs expressed in immune cells are essential in maintaining the physiological functions of immune cells and participate in immune evasion and antitumor immunity by regulating immune cells.

One of the activation hallmarks of oncogenic RAS proteins is the ability to inhibit apoptosis of cancer cells to obtain unlimited proliferation. Oncogenic RAS could have mutations that resist the hydrolytic inhibition triggered by RASGAPs. Although the search for small molecule drugs that could act as equivalents to GAPs to promote RAS-GTP hydrolysis has long been proposed, no optimistic progress has been made. It has been shown that semaphorin 4D acts on the GAP-active receptor Plexin-B1 to inactivate R-Ras and thereby regulate integrin activation and cell migration [137]. Additional examples exist for the regulation of specific GAP activities. For example, the synthetic protein repulsive guidance molecule A (RGMA) receptor neogenin upregulates p120GAP activity, leading to inhibition of Ras and its downstream effector Akt [138]. Inhibition of the function of RASGAPs might also exist in cancer. However, the therapeutic modalities for typically associated NF1 disease are still very difficult, and the current strategies mostly involve inhibition of the RAS/MEK pathway [139]. In summary, although the basic functionality of GAPs is well understood, further studies are necessary to better understand how GAPs regulate biological processes, to correctly understand pathological development and identify therapeutic targets.

## Data Availability

Not applicable.

## Abbreviations

GAP: GTPase-activating protein
RCD: Regulated cell death
GEFs: Guanine nucleotide exchange factors
GDIs: Guanine nucleotide dissociation inhibitors
RGSs: Regulators of G protein signaling
GPCR: G-protein coupled receptor
ACD: Accidental cell death
PCD: Programmed cell death
DISC: Death-inducing signaling complex
TNF: Tumor necrosis factor
TRAIL: TNF-related apoptosis-inducing ligand
RGS3: Regulator of G-protein signaling 3
DLC1: Deleted in liver cancer 1
DAB2IP: DOC-2/DAB2 interacting protein
PITX1: Phosphorylation of pituitary homeobox 1
HCC: Hepatocellular carcinoma
ceRNA: Competing endogenous RNA
PCa: Prostate cancer
ECT2: Epithelial cell transforming sequence 2
YAP: Yes-associated protein
TPR: Translocated promoter region
BLBC: Basal-like breast cancer
LV: Lymphatic vessel
ADCD: Autophagy-dependent cell death
mTOR: Mechanistic target of rapamycin
TMZ: Temozolomide
GBM: Glioblastoma
G3BP1: Ras-GTPase-activating protein-binding protein 1
Yops: *Yersinia* Outer proteins
MC: Mitotic catastrophe
MCT-1: Multiple copies in T-cell malignancy 1
ICD: Immunogenic cell death
CTLs: Cytotoxic T lymphocytes
DAMPs: Damage-associated molecular patterns
APCs: Antigen-presenting cells
DP: Double positive
NF1: Neurofibromatosis type 1
SCF: Stem cell factor
LGG: Low-grade glioma
Rasal1: Ras protein activator-like 1 protein

## References

[1] Wennerberg K, Rossman KL, Der CJ (2005). The Ras superfamily at a glance. J Cell Sci.

[2] Takai Y, Sasaki T, Matozaki T (2001). Small GTP-binding proteins. Physiol Rev.

[3] Bos JL, Rehmann H, Wittinghofer A (2007). GEFs and GAPs: critical elements in the control of small G proteins. Cell.

[4] Cherfils J, Zeghouf M (2013). Regulation of small GTPases by GEFs, GAPs, and GDIs. Physiol Rev.

[5] Ligeti E, Welti S, Scheffzek K (2012). Inhibition and termination of physiological responses by GTPase activating proteins. Physiol Rev.

[6] Ross EM, Wilkie TM (2000). GTPase-activating proteins for heterotrimeric G proteins: regulators of G protein signaling (RGS) and RGS-like proteins. Annu Rev Biochem.

[7] Scheffzek K, Ahmadian MR, Kabsch W, Wiesmuller L, Lautwein A, Schmitz F, Wittinghofer A (1997). The Ras-RasGAP complex: structural basis for GTPase activation and its loss in oncogenic Ras mutants. Science.

[8] Scheffzek K, Shivalingaiah G (2019). Ras-specific GTPase-activating proteins-structures, mechanisms, and interactions. Cold Spring Harb Perspect Med.

[9] Tang D, Kang R, Berghe TV, Vandenabeele P, Kroemer G (2019). The molecular machinery of regulated cell death. Cell Res.

[10] Galluzzi L, Vitale I, Aaronson SA, Abrams JM, Adam D, Agostinis P, Alnemri ES, Altucci L, Amelio I, Andrews DW, Annicchiarico-Petruzzelli M, Antonov AV, Arama E, Baehrecke EH, Barlev NA, Bazan NG, Bernassola F, Bertrand MJM, Bianchi K, Blagosklonny MV, Blomgren K, Borner C, Boya P, Brenner C, Campanella M, Candi E, Carmona-Gutierrez D, Cecconi F, Chan FK, Chandel NS (2018). Molecular mechanisms of cell death: recommendations of the Nomenclature Committee on Cell Death 2018. Cell Death Differ.

[11] Maiuri MC, Zalckvar E, Kimchi A, Kroemer G (2007). Self-eating and self-killing: crosstalk between autophagy and apoptosis. Nat Rev Mol Cell Biol.

[12] Kerr JF (1971). Shrinkage necrosis: a distinct mode of cellular death. J Pathol.

[13] Carneiro BA, El-Deiry WS (2020). Targeting apoptosis in cancer therapy. Nat Rev Clin Oncol.

[14] Czabotar PE, Lessene G, Strasser A, Adams JM (2014). Control of apoptosis by the BCL-2 protein family: implications for physiology and therapy. Nat Rev Mol Cell Biol.

[15] Seyrek K, Ivanisenko NV, Richter M, Hillert LK, Konig C, Lavrik IN (2020). Controlling cell death through post-translational modifications of DED proteins. Trends Cell Biol.

[16] Tai WT, Chen YL, Chu PY, Chen LJ, Hung MH, Shiau CW, Huang JW, Tsai MH, Chen KF (2016). Protein tyrosine phosphatase 1B dephosphorylates PITX1 and regulates p120RasGAP in hepatocellular carcinoma. Hepatology.

[17] Vanli G, Sempoux C, Widmann C (2017). The caspase-3/p120 RasGAP stress-sensing module reduces liver cancer incidence but does not affect overall survival in gamma-irradiated and carcinogen-treated mice. Mol Carcinog.

[18] Yang JY, Michod D, Walicki J, Murphy BM, Kasibhatla S, Martin SJ, Widmann C (2004). Partial cleavage of RasGAP by caspases is required for cell survival in mild stress conditions. Mol Cell Biol.

[19] Lu S, Zhou J, Sun Y, Li N, Miao M, Jiao B, Chen H (2017). The noncoding RNA HOXD-AS1 is a critical regulator of the metastasis and apoptosis phenotype in human hepatocellular carcinoma. Mol Cancer.

[20] Guo X, Xiang C, Zhang Z, Zhang F, Xi T, Zheng L (2018). Displacement of Bax by BMF mediates STARD13 3'UTR-induced breast cancer cells apoptosis in an miRNA-dependent manner. Mol Pharm.

[21] Sánchez-Martín D, Otsuka A, Kabashima K, Ha T, Wang D, Qian X, Lowy DR, Tosato G (2018). Effects of DLC1 deficiency on endothelial cell contact growth inhibition and angiosarcoma progression. J Natl Cancer Inst.

[22] Bellazzo A, Di Minin G, Collavin L (2017). Block one, unleash a hundred. Mechanisms of DAB2IP inactivation in cancer. Cell Death Differ.

[23] Zhou J, Ning Z, Wang B, Yun EJ, Zhang T, Pong RC, Fazli L, Gleave M, Zeng J, Fan J, Wang X, Li L, Hsieh JT, He D, Wu K (2015). DAB2IP loss confers the resistance of prostate cancer to androgen deprivation therapy through activating STAT3 and inhibiting apoptosis. Cell Death Dis.

[24] Yang XM, Cao XY, He P, Li J, Feng MX, Zhang YL, Zhang XL, Wang YH, Yang Q, Zhu L, Nie HZ, Jiang SH, Tian GA, Zhang XX, Liu Q, Ji J, Zhu X, Xia Q, Zhang ZG (2018). Overexpression of Rac GTPase activating protein 1 contributes to proliferation of cancer cells by reducing hippo signaling to promote cytokinesis. Gastroenterology.

[25] Tatsumoto T, Xie X, Blumenthal R, Okamoto I, Miki T (1999). Human ECT2 is an exchange factor for Rho GTPases, phosphorylated in G2/M phases, and involved in cytokinesis. J Cell Biol.

[26] Chen J, Xia H, Zhang X, Karthik S, Pratap SV, Ooi LL, Hong W, Hui KM (2015). ECT2 regulates the Rho/ERK signalling axis to promote early recurrence in human hepatocellular carcinoma. J Hepatol.

[27] Lawson CD, Fan C, Mitin N, Baker NM, George SD, Graham DM, Perou CM, Burridge K, Der CJ, Rossman KL (2016). Rho GTPase transcriptome analysis reveals oncogenic roles for rho GTPase-activating proteins in basal-like breast cancers. Cancer Res.

[28] Lapinski PE, Lubeck BA, Chen D, Doosti A, Zawieja SD, Davis MJ, King PD (2017). RASA1 regulates the function of lymphatic vessel valves in mice. J Clin Invest.

[29] Wang Z, Huang H, He W, Kong B, Hu H, Fan Y, Liao J, Wang L, Mei Y, Liu W, Xiong X, Peng J, Xiao Y, Huang D, Quan D, Li Q, Xiong L, Zhong P, Wang G (2016). Regulator of G-protein signaling 5 protects cardiomyocytes against apoptosis during in vitro cardiac ischemia-reperfusion in mice by inhibiting both JNK1/2 and P38 signaling pathways. Biochem Biophys Res Commun.

[30] Wang H, Fan L, Wang H, Ma X, Du Z (2015). Amyloid beta regulates the expression and function of AIP1. J Mol Neurosci.

[31] Sirohi K, Swarup G (2016). Defects in autophagy caused by glaucoma-associated mutations in optineurin. Exp Eye Res.

[32] Shen HM, Codogno P (2011). Autophagic cell death: Loch Ness monster or endangered species?. Autophagy.

[33] Kriel J, Loos B (2019). The good, the bad and the autophagosome: exploring unanswered questions of autophagy-dependent cell death. Cell Death Differ.

[34] Lindqvist LM, Simon AK, Baehrecke EH (2015). Current questions and possible controversies in autophagy. Cell Death Discov.

[35] Amaravadi R, Kimmelman AC, White E (2016). Recent insights into the function of autophagy in cancer. Genes Dev.

[36] Kimmelman AC, White E (2017). Autophagy and tumor metabolism. Cell Metab.

[37] Kanzawa T, Kondo Y, Ito H, Kondo S, Germano I (2003). Induction of autophagic cell death in malignant glioma cells by arsenic trioxide. Cancer Res.

[38] Dasari SK, Bialik S, Levin-Zaidman S, Levin-Salomon V, Merrill AH, Futerman AH, Kimchi A (2017). Signalome-wide RNAi screen identifies GBA1 as a positive mediator of autophagic cell death. Cell Death Differ.

[39] Zein L, Fulda S, Kogel D, van Wijk SJL (2021). Organelle-specific mechanisms of drug-induced autophagy-dependent cell death. Matrix Biol.

[40] Elgendy M, Sheridan C, Brumatti G, Martin SJ (2011). Oncogenic Ras-induced expression of Noxa and Beclin-1 promotes autophagic cell death and limits clonogenic survival. Mol Cell.

[41] Stenmark H (2009). Rab GTPases as coordinators of vesicle traffic. Nat Rev Mol Cell Biol.

[42] Hyttinen JM, Niittykoski M, Salminen A, Kaarniranta K (2013). Maturation of autophagosomes and endosomes: a key role for Rab7. Biochim Biophys Acta.

[43] Szatmari Z, Sass M (2014). The autophagic roles of Rab small GTPases and their upstream regulators: a review. Autophagy.

[44] Pantoom S, Konstantinidis G, Voss S, Han H, Hofnagel O, Li Z, Wu YW (2020). RAB33B recruits the ATG16L1 complex to the phagophore via a noncanonical RAB binding protein. Autophagy.

[45] Itoh T, Kanno E, Uemura T, Waguri S, Fukuda M (2011). OATL1, a novel autophagosome-resident Rab33B-GAP, regulates autophagosomal maturation. J Cell Biol.

[46] Moskalenko S, Henry DO, Rosse C, Mirey G, Camonis JH, White MA (2002). The exocyst is a Ral effector complex. Nat Cell Biol.

[47] Bodemann BO, Orvedahl A, Cheng T, Ram RR, Ou YH, Formstecher E, Maiti M, Hazelett CC, Wauson EM, Balakireva M, Camonis JH, Yeaman C, Levine B, White MA (2011). RalB and the exocyst mediate the cellular starvation response by direct activation of autophagosome assembly. Cell.

[48] Tracy K, Velentzas PD, Baehrecke EH (2016). Ral GTPase and the exocyst regulate autophagy in a tissue-specific manner. EMBO Rep.

[49] Kim J, Kundu M, Viollet B, Guan KL (2011). AMPK and mTOR regulate autophagy through direct phosphorylation of Ulk1. Nat Cell Biol.

[50] Huang J, Manning BD (2009). A complex interplay between Akt, TSC2 and the two mTOR complexes. Biochem Soc Trans.

[51] Xiang H, Zhang J, Lin C, Zhang L, Liu B, Ouyang L (2020). Targeting autophagy-related protein kinases for potential therapeutic purpose. Acta Pharm Sin B.

[52] Henske EP, Jozwiak S, Kingswood JC, Sampson JR, Thiele EA (2016). Tuberous sclerosis complex. Nat Rev Dis Primers.

[53] Martin TD, Chen XW, Kaplan RE, Saltiel AR, Walker CL, Reiner DJ, Der CJ (2014). Ral and Rheb GTPase activating proteins integrate mTOR and GTPase signaling in aging, autophagy, and tumor cell invasion. Mol Cell.

[54] Yun EJ, Kim S, Hsieh JT, Baek ST (2020). Wnt/beta-catenin signaling pathway induces autophagy-mediated temozolomide-resistance in human glioblastoma. Cell Death Dis.

[55] Liao H, Xiao Y, Hu Y, Xiao Y, Yin Z, Liu L (2015). microRNA-32 induces radioresistance by targeting DAB2IP and regulating autophagy in prostate cancer cells. Oncol Lett.

[56] Zeng X, Kinsella TJ (2008). Mammalian target of rapamycin and S6 kinase 1 positively regulate 6-thioguanine-induced autophagy. Cancer Res.

[57] Yu L, Tumati V, Tseng SF, Hsu FM, Kim DN, Hong D, Hsieh JT, Jacobs C, Kapur P, Saha D (2012). DAB2IP regulates autophagy in prostate cancer in response to combined treatment of radiation and a DNA-PKcs inhibitor. Neoplasia.

[58] Andres-Alonso M, Ammar MR, Butnaru I, Gomes GM, Acuna Sanhueza G, Raman R, Yuanxiang P, Borgmeyer M, Lopez-Rojas J, Raza SA, Brice N, Hausrat TJ, Macharadze T, Diaz-Gonzalez S, Carlton M, Failla AV, Stork O, Schweizer M, Gundelfinger ED, Kneussel M, Spilker C, Karpova A, Kreutz MR (2019). SIPA1L2 controls trafficking and local signaling of TrkB-containing amphisomes at presynaptic terminals. Nat Commun.

[59] Tan EHN, Tang BL (2019). Rab7a and mitophagosome formation. Cells.

[60] Sun M, Luong G, Plastikwala F, Sun Y (2020). Control of Rab7a activity and localization through endosomal type Igamma PIP 5-kinase is required for endosome maturation and lysosome function. FASEB J.

[61] Seaman MNJ, Mukadam AS, Breusegem SY (2018). Inhibition of TBC1D5 activates Rab7a and can enhance the function of the retromer cargo-selective complex. J Cell Sci.

[62] Nehammer C, Ejlerskov P, Gopal S, Handley A, Ng L, Moreira P, Lee H, Issazadeh-Navikas S, Rubinsztein DC, Pocock R (2019). Interferon-beta-induced miR-1 alleviates toxic protein accumulation by controlling autophagy. Elife.

[63] Ejlerskov P, Rubinsztein DC, Pocock R (2020). IFNB/interferon-beta regulates autophagy via a MIR1-TBC1D15-RAB7 pathway. Autophagy.

[64] Lin M, Liu H, Xiong Q, Niu H, Cheng Z, Yamamoto A, Rikihisa Y (2016). Ehrlichia secretes Etf-1 to induce autophagy and capture nutrients for its growth through RAB5 and class III phosphatidylinositol 3-kinase. Autophagy.

[65] Minowa-Nozawa A, Nozawa T, Okamoto-Furuta K, Kohda H, Nakagawa I (2017). Rab35 GTPase recruits NDP52 to autophagy targets. EMBO J.

[66] Sidjanin DJ, Park AK, Ronchetti A, Martins J, Jackson WT (2016). TBC1D20 mediates autophagy as a key regulator of autophagosome maturation. Autophagy.

[67] Roy S, Leidal AM, Ye J, Ronen SM, Debnath J (2017). Autophagy-dependent shuttling of TBC1D5 controls plasma membrane translocation of GLUT1 and glucose uptake. Mol Cell.

[68] Conrad M, Pratt DA (2019). The chemical basis of ferroptosis. Nat Chem Biol.

[69] Dixon SJ, Lemberg KM, Lamprecht MR, Skouta R, Zaitsev EM, Gleason CE, Patel DN, Bauer AJ, Cantley AM, Yang WS, Morrison B, Stockwell BR (2012). Ferroptosis: an iron-dependent form of nonapoptotic cell death. Cell.

[70] Xie Y, Hou W, Song X, Yu Y, Huang J, Sun X, Kang R, Tang D (2016). Ferroptosis: process and function. Cell Death Differ.

[71] Chen X, Kang R, Kroemer G, Tang D (2021). Broadening horizons: the role of ferroptosis in cancer. Nat Rev Clin Oncol.

[72] Yagoda N, von Rechenberg M, Zaganjor E, Bauer AJ, Yang WS, Fridman DJ, Wolpaw AJ, Smukste I, Peltier JM, Boniface JJ, Smith R, Lessnick SL, Sahasrabudhe S, Stockwell BR (2007). RAS-RAF-MEK-dependent oxidative cell death involving voltage-dependent anion channels. Nature.

[73] Zhou B, Liu J, Kang R, Klionsky DJ, Kroemer G, Tang D (2020). Ferroptosis is a type of autophagy-dependent cell death. Semin Cancer Biol.

[74] Bai Y, Meng L, Han L, Jia Y, Zhao Y, Gao H, Kang R, Wang X, Tang D, Dai E (2019). Lipid storage and lipophagy regulates ferroptosis. Biochem Biophys Res Commun.

[75] Toyofuku T, Morimoto K, Sasawatari S, Kumanogoh A (2015). Leucine-rich repeat kinase 1 regulates autophagy through turning on TBC1D2-dependent Rab7 inactivation. Mol Cell Biol.

[76] Mao C, Wang X, Liu Y, Wang M, Yan B, Jiang Y, Shi Y, Shen Y, Liu X, Lai W, Yang R, Xiao D, Cheng Y, Liu S, Zhou H, Cao Y, Yu W, Muegge K, Yu H, Tao Y (2018). A G3BP1-interacting lncRNA promotes ferroptosis and apoptosis in cancer via nuclear sequestration of p53. Cancer Res.

[77] Chen KW, Demarco B, Broz P (2020). Beyond inflammasomes: emerging function of gasdermins during apoptosis and NETosis. EMBO J.

[78] McKenzie BA, Dixit VM, Power C (2020). Fiery cell death: pyroptosis in the central nervous system. Trends Neurosci.

[79] Medici NP, Rashid M, Bliska JB (2019). Characterization of pyrin dephosphorylation and inflammasome activation in macrophages as triggered by the yersinia effectors YopE and YopT. Infect Immun.

[80] Overholtzer M, Mailleux AA, Mouneimne G, Normand G, Schnitt SJ, King RW, Cibas ES, Brugge JS (2007). A nonapoptotic cell death process, entosis, that occurs by cell-in-cell invasion. Cell.

[81] White E (2007). Entosis: it's a cell-eat-cell world. Cell.

[82] Florey O, Kim SE, Sandoval CP, Haynes CM, Overholtzer M (2011). Autophagy machinery mediates macroendocytic processing and entotic cell death by targeting single membranes. Nat Cell Biol.

[83] Krishna S, Overholtzer M (2016). Mechanisms and consequences of entosis. Cell Mol Life Sci.

[84] Sun Q, Cibas ES, Huang H, Hodgson L, Overholtzer M (2014). Induction of entosis by epithelial cadherin expression. Cell Res.

[85] Zeng C, Zeng B, Dong C, Liu J, Xing F (2020). Rho-ROCK signaling mediates entotic cell death in tumor. Cell Death Discov.

[86] Vitale I, Galluzzi L, Castedo M, Kroemer G (2011). Mitotic catastrophe: a mechanism for avoiding genomic instability. Nat Rev Mol Cell Biol.

[87] Castedo M, Perfettini JL, Roumier T, Andreau K, Medema R, Kroemer G (2004). Cell death by mitotic catastrophe: a molecular definition. Oncogene.

[88] Castedo M, Coquelle A, Vivet S, Vitale I, Kauffmann A, Dessen P, Pequignot MO, Casares N, Valent A, Mouhamad S, Schmitt E, Modjtahedi N, Vainchenker W, Zitvogel L, Lazar V, Garrido C, Kroemer G (2006). Apoptosis regulation in tetraploid cancer cells. EMBO J.

[89] Zhou X, Kim SH, Shen L, Lee HJ, Chen C (2014). Induction of mitotic catastrophe by PKC inhibition in Nf1-deficient cells. Cell Cycle.

[90] Wu MH, Chen YA, Chen HH, Chang KW, Chang IS, Wang LH, Hsu HL (2014). MCT-1 expression and PTEN deficiency synergistically promote neoplastic multinucleation through the Src/p190B signaling activation. Oncogene.

[91] Hashizume C, Kobayashi A, Wong RW (2013). Down-modulation of nucleoporin RanBP2/Nup358 impaired chromosomal alignment and induced mitotic catastrophe. Cell Death Dis.

[92] Nirmala JG, Lopus M (2019). Cell death mechanisms in eukaryotes. Cell Biol Toxicol.

[93] Chi S, Kitanaka C, Noguchi K, Mochizuki T, Nagashima Y, Shirouzu M, Fujita H, Yoshida M, Chen W, Asai A, Himeno M, Yokoyama S, Kuchino Y (1999). Oncogenic Ras triggers cell suicide through the activation of a caspase-independent cell death program in human cancer cells. Oncogene.

[94] Bhanot H, Young AM, Overmeyer JH, Maltese WA (2010). Induction of nonapoptotic cell death by activated Ras requires inverse regulation of Rac1 and Arf6. Mol Cancer Res.

[95] Maltese WA, Overmeyer JH (2014). Methuosis: nonapoptotic cell death associated with vacuolization of macropinosome and endosome compartments. Am J Pathol.

[96] Porat-Shliom N, Kloog Y, Donaldson JG (2008). A unique platform for H-Ras signaling involving clathrin-independent endocytosis. Mol Biol Cell.

[97] Green DR, Ferguson T, Zitvogel L, Kroemer G (2009). Immunogenic and tolerogenic cell death. Nat Rev Immunol.

[98] Galluzzi L, Vitale I, Warren S, Adjemian S, Agostinis P, Martinez AB, Chan TA, Coukos G, Demaria S, Deutsch E, Draganov D, Edelson RL, Formenti SC, Fucikova J, Gabriele L, Gaipl US, Gameiro SR, Garg AD, Golden E, Han J, Harrington KJ, Hemminki A, Hodge JW, Hossain DMS, Illidge T, Karin M, Kaufman HL, Kepp O, Kroemer G, Lasarte JJ (2020). Consensus guidelines for the definition, detection and interpretation of immunogenic cell death. J Immunother Cancer.

[99] Kroemer G, Galluzzi L, Kepp O, Zitvogel L (2013). Immunogenic cell death in cancer therapy. Annu Rev Immunol.

[100] Casares N, Pequignot MO, Tesniere A, Ghiringhelli F, Roux S, Chaput N, Schmitt E, Hamai A, Hervas-Stubbs S, Obeid M, Coutant F, Metivier D, Pichard E, Aucouturier P, Pierron G, Garrido C, Zitvogel L, Kroemer G (2005). Caspase-dependent immunogenicity of doxorubicin-induced tumor cell death. J Exp Med.

[101] Dudek AM, Garg AD, Krysko DV, De Ruysscher D, Agostinis P (2013). Inducers of immunogenic cancer cell death. Cytokine Growth Factor Rev.

[102] Wilson AL, Plebanski M, Stephens AN (2018). New trends in anti-cancer therapy: combining conventional chemotherapeutics with novel immunomodulators. Curr Med Chem.

[103] Jeong SD, Jung BK, Ahn HM, Lee D, Ha J, Noh I, Yun CO, Kim YC (2021). Immunogenic cell death inducing fluorinated mitochondria-disrupting helical polypeptide synergizes with PD-L1 immune checkpoint blockade. Adv Sci (Weinh).

[104] Mathew M, Enzler T, Shu CA, Rizvi NA (2018). Combining chemotherapy with PD-1 blockade in NSCLC. Pharmacol Ther.

[105] Duan X, Chan C, Lin W (2019). Nanoparticle-mediated immunogenic cell death enables and potentiates cancer immunotherapy. Angew Chem Int Ed Engl.

[106] Yang S, Sun IC, Hwang HS, Shim MK, Yoon HY, Kim K (2021). Rediscovery of nanoparticle-based therapeutics: boosting immunogenic cell death for potential application in cancer immunotherapy. J Mater Chem B.

[107] Legrand AJ, Konstantinou M, Goode EF, Meier P (2019). The diversification of cell death and immunity: memento Mori. Mol Cell.

[108] Messmer MN, Snyder AG, Oberst A (2019). Comparing the effects of different cell death programs in tumor progression and immunotherapy. Cell Death Differ.

[109] Tang R, Xu J, Zhang B, Liu J, Liang C, Hua J, Meng Q, Yu X, Shi S (2020). Ferroptosis, necroptosis, and pyroptosis in anticancer immunity. J Hematol Oncol.

[110] Wang W, Green M, Choi JE, Gijon M, Kennedy PD, Johnson JK, Liao P, Lang X, Kryczek I, Sell A, Xia H, Zhou J, Li G, Li J, Li W, Wei S, Vatan L, Zhang H, Szeliga W, Gu W, Liu R, Lawrence TS, Lamb C, Tanno Y, Cieslik M, Stone E, Georgiou G, Chan TA, Chinnaiyan A, Zou W (2019). CD8(+) T cells regulate tumour ferroptosis during cancer immunotherapy. Nature.

[111] Alberola-Ila J, Hernandez-Hoyos G (2003). The Ras/MAPK cascade and the control of positive selection. Immunol Rev.

[112] Fischer AM, Katayama CD, Pages G, Pouyssegur J, Hedrick SM (2005). The role of erk1 and erk2 in multiple stages of T cell development. Immunity.

[113] Lapinski PE, Qiao Y, Chang CH, King PD (2011). A role for p120 RasGAP in thymocyte positive selection and survival of naive T cells. J Immunol.

[114] Ito-Kureha T, Miyao T, Nishijima S, Suzuki T, Koizumi SI, Villar-Briones A, Takahashi A, Akiyama N, Morita M, Naguro I, Ishikawa H, Ichijo H, Akiyama T, Yamamoto T (2020). The CCR4-NOT deadenylase complex safeguards thymic positive selection by down-regulating aberrant pro-apoptotic gene expression. Nat Commun.

[115] Oliver JA, Lapinski PE, Lubeck BA, Turner JS, Parada LF, Zhu Y, King PD (2013). The Ras GTPase-activating protein neurofibromin 1 promotes the positive selection of thymocytes. Mol Immunol.

[116] David MD, Petit D, Bertoglio J (2014). The RhoGAP ARHGAP19 controls cytokinesis and chromosome segregation in T lymphocytes. J Cell Sci.

[117] He L, Valignat MP, Zhang L, Gelard L, Zhang F, Le Guen V, Audebert S, Camoin L, Fossum E, Bogen B, Wang H, Henri S, Roncagalli R, Theodoly O, Liang Y, Malissen M, Malissen B (2021). ARHGAP45 controls naive T- and B-cell entry into lymph nodes and T-cell progenitor thymus seeding. EMBO Rep.

[118] Patino-Lopez G, Dong X, Ben-Aissa K, Bernot KM, Itoh T, Fukuda M, Kruhlak MJ, Samelson LE, Shaw S (2008). Rab35 and its GAP EPI64C in T cells regulate receptor recycling and immunological synapse formation. J Biol Chem.

[119] Caron E, Hall A (1998). Identification of two distinct mechanisms of phagocytosis controlled by different Rho GTPases. Science.

[120] Massol P, Montcourrier P, Guillemot JC, Chavrier P (1998). Fc receptor-mediated phagocytosis requires CDC42 and Rac1. EMBO J.

[121] Schlam D, Bagshaw RD, Freeman SA, Collins RF, Pawson T, Fairn GD, Grinstein S (2015). Phosphoinositide 3-kinase enables phagocytosis of large particles by terminating actin assembly through Rac/Cdc42 GTPase-activating proteins. Nat Commun.

[122] Pixley FJ, Xiong Y, Yu RY, Sahai EA, Stanley ER, Ye BH (2005). BCL6 suppresses RhoA activity to alter macrophage morphology and motility. J Cell Sci.

[123] Hemkemeyer SA, Vollmer V, Schwarz V, Lohmann B, Honnert U, Taha M, Schnittler HJ, Bahler M (2021). Local Myo9b RhoGAP activity regulates cell motility. J Biol Chem.

[124] Csepanyi-Komi R, Pasztor M, Bartos B, Ligeti E (2018). The neglected terminators: Rho family GAPs in neutrophils. Eur J Clin Invest.

[125] Basu TN, Gutmann DH, Fletcher JA, Glover TW, Collins FS, Downward J (1992). Aberrant regulation of ras proteins in malignant tumour cells from type 1 neurofibromatosis patients. Nature.

[126] Le LQ, Parada LF (2007). Tumor microenvironment and neurofibromatosis type I: connecting the GAPs. Oncogene.

[127] Zhu Y, Ghosh P, Charnay P, Burns DK, Parada LF (2002). Neurofibromas in NF1: Schwann cell origin and role of tumor environment. Science.

[128] Yang FC, Ingram DA, Chen S, Hingtgen CM, Ratner N, Monk KR, Clegg T, White H, Mead L, Wenning MJ, Williams DA, Kapur R, Atkinson SJ, Clapp DW (2003). Neurofibromin-deficient Schwann cells secrete a potent migratory stimulus for Nf1+/- mast cells. J Clin Invest.

[129] Chen S, Burgin S, McDaniel A, Li X, Yuan J, Chen M, Khalaf W, Clapp DW, Yang FC (2010). Nf1-/- Schwann cell-conditioned medium modulates mast cell degranulation by c-Kit-mediated hyperactivation of phosphatidylinositol 3-kinase. Am J Pathol.

[130] Staser K, Yang FC, Clapp DW (2010). Mast cells and the neurofibroma microenvironment. Blood.

[131] Guo X, Pan Y, Xiong M, Sanapala S, Anastasaki C, Cobb O, Dahiya S, Gutmann DH (2020). Midkine activation of CD8(+) T cells establishes a neuron-immune-cancer axis responsible for low-grade glioma growth. Nat Commun.

[132] Gangoso E, Southgate B, Bradley L, Rus S, Galvez-Cancino F, McGivern N, Guc E, Kapourani CA, Byron A, Ferguson KM, Alfazema N, Morrison G, Grant V, Blin C, Sou I, Marques-Torrejon MA, Conde L, Parrinello S, Herrero J, Beck S, Brandner S, Brennan PM, Bertone P, Pollard JW, Quezada SA, Sproul D, Frame MC, Serrels A, Pollard SM (2021). Glioblastomas acquire myeloid-affiliated transcriptional programs via epigenetic immunoediting to elicit immune evasion. Cell.

[133] Brosseau JP, Liao CP, Wang Y, Ramani V, Vandergriff T, Lee M, Patel A, Ariizumi K, Le LQ (2018). NF1 heterozygosity fosters de novo tumorigenesis but impairs malignant transformation. Nat Commun.

[134] Thaker YR, Raab M, Strebhardt K, Rudd CE (2019). GTPase-activating protein Rasal1 associates with ZAP-70 of the TCR and negatively regulates T-cell tumor immunity. Nat Commun.

[135] Xie Z, Chan EC, Druey KM (2016). R4 regulator of G protein signaling (RGS) proteins in inflammation and immunity. AAPS J.

[136] Huang D, Chen X, Zeng X, Lao L, Li J, Xing Y, Lu Y, Ouyang Q, Chen J, Yang L, Su F, Yao H, Liu Q, Su S, Song E (2021). Targeting regulator of G protein signaling 1 in tumor-specific T cells enhances their trafficking to breast cancer. Nat Immunol.

[137] Oinuma I, Katoh H, Negishi M (2006). Semaphorin 4D/Plexin-B1-mediated R-Ras GAP activity inhibits cell migration by regulating beta(1) integrin activity. J Cell Biol.

[138] Endo M, Yamashita T (2009). Inactivation of Ras by p120GAP via focal adhesion kinase dephosphorylation mediates RGMa-induced growth cone collapse. J Neurosci.

[139] Walker JA, Upadhyaya M (2018). Emerging therapeutic targets for neurofibromatosis type 1. Expert Opin Ther Targets.

